# Robust Single Trial Identification of Conscious Percepts Triggered by Sensory Events of Variable Saliency

**DOI:** 10.1371/journal.pone.0086201

**Published:** 2014-01-23

**Authors:** Marta Teixeira, Gabriel Pires, Miguel Raimundo, Sérgio Nascimento, Vasco Almeida, Miguel Castelo-Branco

**Affiliations:** 1 Visual Neuroscience Laboratory, IBILI - Institute for Biomedical Imaging and Life Sciences, University of Coimbra, Coimbra, Portugal; 2 Institute for Nuclear Sciences Applied to Health (ICNAS), Brain Imaging Network of Portugal, University of Coimbra, Coimbra, Portugal; 3 Institute for Systems and Robotics (ISR), University of Coimbra, Coimbra, Portugal; 4 Department of Physics, University of Minho, Braga, Portugal; 5 Department of Physics, University of Beira Interior, Covilhã, Portugal; French National Centre for Scientific Research, France

## Abstract

The neural correlates of visual awareness are elusive because of its fleeting nature. Here we have addressed this issue by using single trial statistical “brain reading” of neurophysiological event related (ERP) signatures of conscious perception of visual attributes with different levels of saliency. Behavioral reports were taken at every trial in 4 experiments addressing conscious access to color, luminance, and local phase offset cues. We found that single trial neurophysiological signatures of target presence can be observed around 300 ms at central parietal sites. Such signatures are significantly related with conscious perception, and their probability is related to sensory saliency levels. These findings identify a general neural correlate of conscious perception at the single trial level, since conscious perception can be decoded as such independently of stimulus salience and fluctuations of threshold levels. This approach can be generalized to successfully detect target presence in other individuals.

## Introduction

Event related cognitive studies are in general based on the interpretation of average responses to many repetitions of a given stimulus or condition [1]–[4]. This renders the direct identification of the neural correlates of visual awareness difficult, because levels of awareness are often fleeting [5]. Inference from brain signals based on global averages cannot address the significance of single cognitive or sensory events. Investigation of perceptual awareness phenomena at the single trial level has to overcome the issue of low signal to noise ratios, and tools for this endeavor are now available [6], [7].

The direct identification of the neural correlates of single perceptual representation moments would allow the understanding of their neurophysiological underpinnings and if detected in a statistically robust manner, they might even be used to predict target presence in other individuals. In other words, then one could generalize across subjects the neurophysiological signatures of such representations.

Classification of single trials has traditionally been difficult, especially because data driven methods have been rarely used in this context. Here, we have departed from the average based traditional model and used oddball paradigms, because they involve rare, unpredictable perceptual events [6], [7]. Although oddball paradigms often elicit a P300 signal that is believed to relate to perceived stimulus changes, this work is not, by its nature, focused on this component. This is because P300 does vary across subjects and conditions both in term of amplitude and latencies [1]–[3], [8]–[12]. Moreover the fact that average responses based on many trials might look “always the same” does not imply that single trial responses are similar. For this reason, the P300 reflects an average measure that can only be indirectly related to conscious, explicit perception [8], [9], [11], [13], [14]. In fact, it remains an open question whether the P300 signals conscious perception [15]–[17]. This question can only be answered at the single trial level and represents one of the major aims of this work. Since our approach is data driven, we could go beyond previous studies of the neural underpinnings of the classical P300 wave and target the identification of individual change detection events and their relation to conscious perception. Single trial analysis is a major undertaking given the challenge to classify neurophysiological events and relate them with perception embedded in noise. Directly pinpointing individual perceptual events is not possible with average analysis.

In any case, centro-parietal average P300 components are generated whenever a discrimination task is required between at least two classes of events: a frequent (Standard) and an infrequent/rare one (Target) [1], [2], [12], [18], [19], and were therefore also measured in our study. For single trial analysis we generated waveform templates for our data driven classification approach [6], [7]. However, this does not imply that the temporally coincident features used to classify the signal belong to the classical P300.

**Figure 1:**
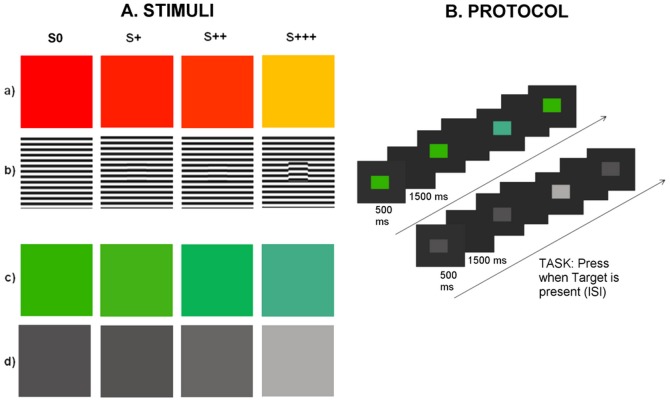
Examples of frequent standard stimuli (leftmost) and rare detection targets (3 rightmost). Manipulation of physical and perceptual saliency was imposed in 4 different experiments: A. Mixed color/luminance (a) and phase offset (b) chrominance (c) and luminance (d). Subjects were required to report the presence of a target as manifested by detectable categorical deviances from the standard stimuli. Stimulus physical properties are described in the text (appearance maybe different from the actual display due to issues such as color rendering, size and resolution). B. Experimental protocol (identical in all experiments and therefore only shown for the monotonic saliency manipulations of chrominance and luminance). Oddball: for each experimental condition - 20 blocks of 50 trials with sequentially displayed Standards (S0) and randomly allocated Targets (S+,S++ and S+++), with a target frequency ratio of 1∶16 (47 ST/3T). Total target presentation number: 20. Stimuli were displayed for 500 ms, with an Inter Stimulus Interval (ISI) of 1500 ms. ISI: grey blank screen with identical colorimetric properties to stimuli's background. Task: press a button during the ISI when Target is present.

In order to ensure variations in perceptual awareness independently of the type of visual attribute, we manipulated multiple independent feature dimensions: color, luminance and phase offset, at different saliency levels (Fig. 1 A).

The roles of distinct saliency levels in visual awareness were tested and replicated in separate experimental sessions: Experimental session I, testing the effects on Color/Luminance and Phase Offset saliency levels; Experimental session II, attempting explicit separation of Luminance and Chrominance effects.

These manipulations ensured that our stimulus conditions were such, that at different trials, perception was above or below threshold.

We identified a neural correlate of conscious processing of single trial sensory events. This detectable marker of conscious access predicted across trial neural detection of other target events with high statistical significance and generalized for detection significantly above chance levels across subjects and experiments.

## Materials and Methods

### Ethics Statement

This study and all the procedures were reviewed and approved by the Ethics Committee of the Faculty of Medicine of the University of Coimbra (Comissão de Ética da Faculdade de Medicina da Universidade de Coimbra). The study was conducted in accordance with the tenets of the Declaration of Helsinki and all participants signed a written informed consent.

### 1. Subjects

In each of the 4 experiments we recruited 10 participants (4 independent replications of the main effect across visual attributes). These were recruited within the University of Coimbra students' population. Six males and four females participated in Experimental session I, with ages between 22 and 29 years old (Mean = 25; SD = 3.6) and normal to corrected Visual Acuity (VA) 20/20. For Experimental session II, five males and five females participated, with ages between 22 and 29 YO (Mean = 25; SD = 2,3) and normal to corrected VA 20/20. One subject was excluded from this set due to corrupted data. No participants had history of neurological or mental disorders. Subjects were seated comfortably in a chair with armrest to minimize muscular artifacts.

### 2. Stimuli and Task

For all conditions, saliency levels were manipulated according to three levels of intensity deviance from a standard stimulus (S0): minimal deviance (S+); intermediate level deviance (S++); and large saliency deviance – yielding a clearly popping out salient stimulus (S+++), (Fig. 1 A, for details see below).

The experiments were performed in a darkened room. Stimuli were displayed in a 19 inch Cathode Ray Tube (CRT) color monitor (Mitsubishi Diamond Digital), with a resolution of 1280×1024 pixels and refresh rate of 85 Hz. Monitor calibration of color and luminance was carried out with a SpectraColorimeter PR-650 from Photo Research. Gamma correction of the monitor output was achieved via software look-up tables. Cone absorptions were calculated for the phosphor spectral profiles using the Smith & Pokorny (1972, 1975) [20], [21] spectral sensitivities. Viewing distance was of 60 cm.

At the beginning of each session, participants were instructed to press a button on a response box whenever they would detect a target (whether it was a change in color, luminance or phase offset). Motor response was given during the Inter Stimuli Interval (ISI) (to avoid P3 signal contamination by motor components). Vertical and horizontal electrooculograms were used to monitor and reject ocular artifacts before averaging procedures and classification (see also our papers [6] and [7], describing how this issue is dealt with in P300-based BCI experiments). About every 5 minutes, there was a pause for participants to rest.

#### Experimental Session I

Stimuli were generated with MatLab (version 7.3.0.) and presented with the stimulation software STIM2 (Compumedics Neuroscan, El Paso TX, USA). Standards and Targets (10×10 visual angle) consisted of colored (experiment I) or offset phased grating squared patches (experiment II), presented against a grey background with a luminance of 17.3 cd/m^2^, and chromaticity (x,y) = (0.285, 0.284) expressed in the CIE 1931 color space, (Fig. 1 A).

In experiment I, Color/Luminance Targets and Standards were displayed with the following luminance (Y) and chromaticities CIE (*x*, *y*): (S+) Y = 26.9 cd/m^2^ (x,y) = (0.619, 0.338); (S++) Y = 31.3cd/m^2^ (x,y) = (0.592, 0.361); (S+++) Y = 60.3cd/m^2^ (x,y) = (0.478, 0.450); (S0) Y = 26 cd/m^2^ (x,y) = (0.621, 0.337), (Fig. 1 A (a)).

In experiment II, Phase Offset Targets and Standards were displayed manipulating patch center phase offset from standard pi = 0: (S+) pi/200 Rad; (S++) pi/50 Rad; (S+++); pi/2Rad; (S0) no phase offset, (Fig. 1 A (b)). The rationale for this choice of phase offset levels is justified in previous psychophysical work [22]. The centrally segregated offset figure subtended 2×2 degrees of visual angle.

Psychometric curves vary across subjects and to have stimulus levels identical for the whole group, we used a non-parametric monotonic saliency increase approach based on the available literature (as justified for example in [22] for phase offset levels). We note that S+ was under threshold and therefore subliminal. Manipulations of salience were made in ordinal rank steps of increasing deviance from Standard S0. Targets featured increasing levels of physical distance from each of the correspondent Standards. The actual physical parameter distance was therefore manipulated monotonically. The distance between S++ and S+++ targets was larger than between S++ and S0, although this was not necessarily apparent in some of the behavioral data. This is to be expected because once stimuli become supraliminal their detection rate becomes similar but not the respective appearance and saliency. A difference was predicted to be reflected in ERP amplitude and ERP reaction time data (see results confirming this prediction). We also ensured that there was not an unbalance between standard/target classes as a function of saliency. Conditions S+, S++, S+++, all had an equal likelihood of 20/940 = 1/47. Models of training/classification used these ratios.

#### Experimental Session II

For experimental session II, stimuli were generated and presented with Matlab (version 7.3.0), using the Psychophysics Toolbox Version 3 (PTB-3) [23]–[25]. Stimuli consisted of 10×10 degrees of visual angle chromatic (experiment III) or achromatic squared patches (experiment IV), centrally presented against a grey background of colorimetric coordinates of Y = 30 cd/m^2^, (x,y) = (0.285,0.284), (Fig. 1 A).

For the chromatic feature (experiment III), saliency manipulation was performed in the Macleod-Boynton (1979) isoluminant color space, keeping a constant luminance of 30 cd/m^2^. Target and Standards were assigned colorimetric coordinates in lms color space of: (S+) lms(0.1192,0.2066,0.0911); (S++) lms(0.1002,0.1948,0.1216); (S+++) lms(0.1161,0.1958,0.2020); (S0) lms(0.1093,0.2020,0.0888), (Fig. 1 A (c)).

For the luminance feature (experiment IV), Targets and Standards were displayed with the following luminance levels: (S+) 5.8201 cd/m^2^; (S++) 10.2485 cd/m^2^; (S+++) 40.2957 cd/m^2^; (S0) 5,2714 cd/m^2^, (Fig. 1 A (d)).

### 3. Protocol

In our oddball block design [1]–[3], [12] (Fig. 1 B), series of frequent (Standard) stimuli were interspersed with rare stimuli (Target), with a target frequency ratio of 1∶16 (target-to-target frequency ratio that elicits larger P300 amplitudes over midline electrodes) and associated occurrence probability of 6% [26].

20 blocks of 50 trials comprising the sequentially arranged STANDARDS and randomly allocated TARGETS, were displayed for each condition. Target position within blocks was counterbalanced and visual feature conditions were interleaved (color/luminance and phase offset - experimental session I; chrominance and luminance - experimental session II). Each Target saliency level was presented 20 times (each feature condition). Stimuli were displayed for 500 ms, with an Inter Stimulus Interval (ISI) of 1500 ms, consisted of a grey blank slide with identical colorimetric properties to stimuli's background (Fig. 1 B). Each block had duration of 1.67 min. For every 2 runs (1 block of each experiment), there was a break for participants to rest. Pause duration was controlled by the participants.

### 4. Eeg Recording and Analysis

ERPs were recorded using a SynAmps2 electrode array of 64 Ag/AgCl electrodes. Scalp electrodes were referenced to CZ and offline recalculated to linked earlobes. Vertical and horizontal electrooculograms were used to monitor and reject ocular artifacts. Cortical electrode impedances did not exceed 5 kΩ, and signal was digitized at a sampling rate of 1000 Hz, on-line filtered at 200 Hz low pass.

Artifact rejection and averaging was done off-line. Analysis was performed with Scan 4.3 Edit (Compumedics Neuroscan, USA). Continuous EEG signals were bandpass filtered with cutoff frequencies of 0.5 and 30 Hz. All the filtering was performed using the Zero Phase Shift options available in Scan 4.3. Data were segmented into epochs spanning from −100 ms previous to stimulus onset to 600 ms after, sorted by the stimulus target level and baseline corrected to pre-stimulus interval (by subtraction of the average pre-stimulus voltage). Artifact rejection levels were set at ±50 µV and automatically removed.

Epochs were averaged for each stimulus level. Peak responses were measured on averaged waveforms by defining the amplitude voltage as the difference between baseline and the most positive going peak from 250 ms to 600 ms post-stimulus, at the midline parietal electrode location (PZ), where the P300 signal is maximal [2], [12], [19]. The resultant values were then exported to SPSS software and statistical analyses were performed.

### 5. Single Trial Classification

Waveform detection at a single-trial level is a challenging task that requires the use of efficient signal processing and classification methods. We used an approach based on the combination of a statistical spatial filter and a Fisher's linear discriminant (FLD) classifier [27], (as described in Pires 2011, [6]). This methodology has been applied in the context of P300-based brain-computer interfaces, showing performance levels above state-of-the-art [6], [7]. Succinctly, consider the input space of an epoch *k*, represented by 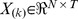, where *N* is the number of channels and *T* is the number of time samples contained in the time window [200 600] ms of the epoch. Based on statistical models of target and non-target epochs, the multichannel input space is transformed into a projection 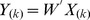 with a much higher signal-to-noise ratio, where 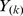 is of dimension 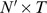, with 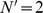, and 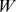 is the spatial filter. The two projections are concatenated and classified with FLD. Classification performance measures for target detection were obtained through leave-one-out (LOO) cross validation technique.

The LOO cross validation approach always uses the proportional amount of data of the stimulus classes according to saliency, covering all available samples. We have actually used S0 (standard) as a benchmark baseline for each binary classification (standard vs. target). The balanced accuracy measure that we used ensures that there is no biasing effect. This effect could indeed occur if we used the “standard accuracy” to measure the classification performance. This happens because the classifier can be simply taking advantage of the imbalance data sets transforming a chance level into a high classification accuracy. False classification rate is taken into account in the measures of specificity, sensitivity, and summarized in fact in the balanced accuracy measure.

## Results

### 1. Behavioral Results –Perceptual Events at the Single Trial Level

**Figure 2:**
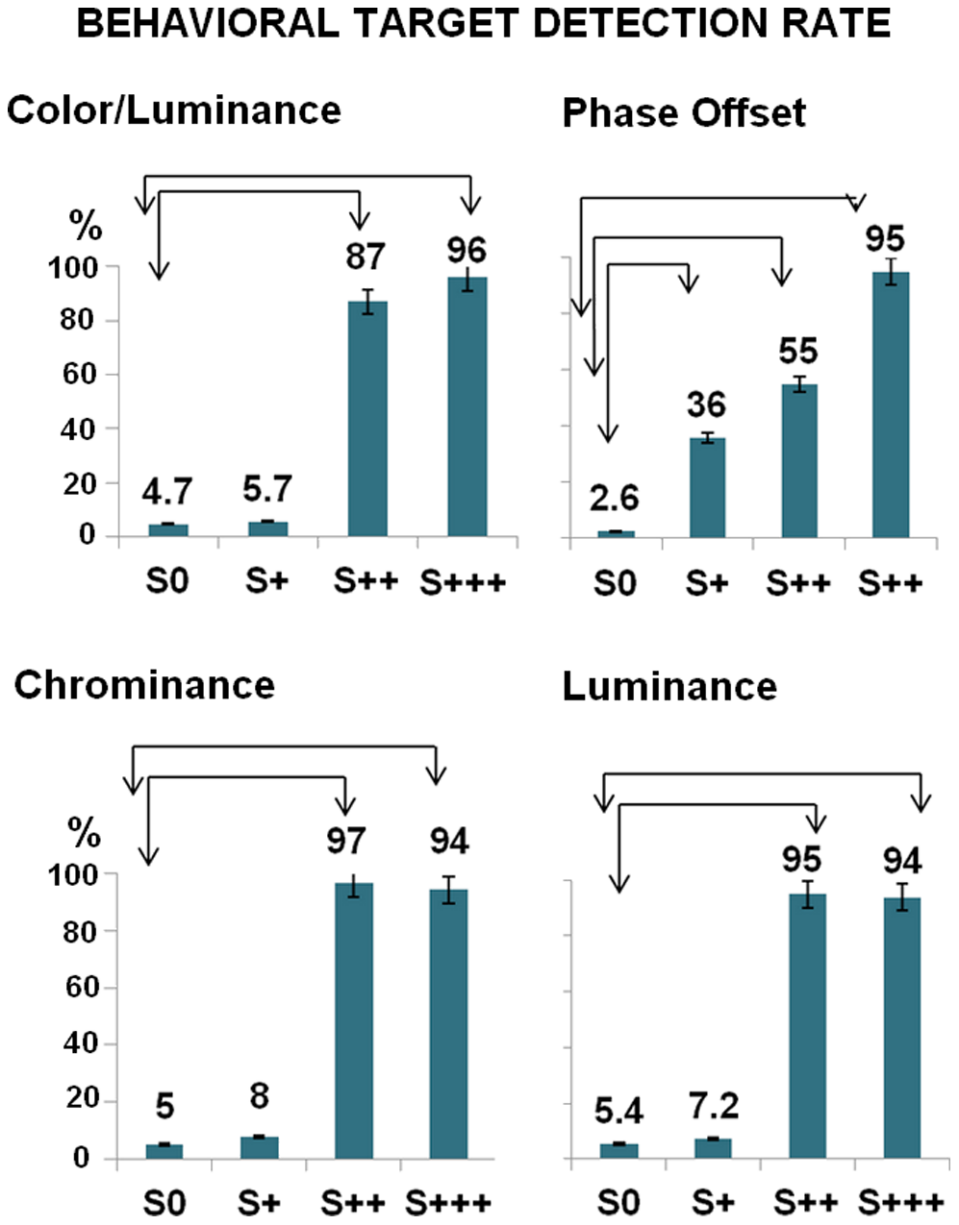
Participant's behavioral Target detection rates (all 4 experiments) and classification of behavioral subliminal vs. supraliminal levels. Note that S+ (saliency level 1) is not separable from S0 (standard reference) from the visual awareness point of view, and can therefore be classified as a subliminal condition, except for the phase offset experiment. Accordingly, no differences were found between both S0 and S+ target detection proportions (binomial distribution test) except for the latter condition. In other words S+ did not elicit responses beyond the guessing the rate (which was very low and similar to the probability of the rare target occurrence). Arrow intervals depict comparisons between S0 and all other conditions (significant at the 0.05 level). Bars depict confidence intervals for the sample means at 95%.

Figure 2 shows group mean percentages of target detection for each experiment (labeled S+, S++ and S+++ according to increasing saliency levels, as defined by increasing physical difference from a standard reference stimulus, S0 being the standard (for details see Methods). S+ proved to be a frequently undetectable stimulus (indistinguishable from S0, in contrast to S++ and S+++). In order to better access the level of subjects' awareness of target saliency manipulation, in addition to the chance probability of detection, we estimated a “guessing rate” for each experiment. This measure was calculated based on subjects' false positive (FP) responses (detection) to standard stimuli (Guessing rate = FP_(false positive)_/(FP_(false positive)_+TN_(true negative)_), (Table S1 in File S1). These guessing rates were then used as null hypothesis proportions in group experiment one-sample binomial statistics, against which target detection proportions (true positives proportion) were compared (Table S2 in File S1).

Participants detected S++ and S+++ saliency manipulations above the guessing rate, but not for the S+ level, except for the phase offset condition (Table S2 in File S1). S+ conditions were therefore perceived as indistinguishable from S0 and therefore out of participants' awareness, except for the latter condition.

This analysis was replicated even when calculating individual guessing rates. Within subject S+ target discrimination from standard is shown in Table S7 in File S1. Except for the phase offset experiment, no differences were found between S+ and Standard S0 detection proportions (Table S7 in File S1). S+ conditions were therefore subliminal, eliciting similar perceptual behavior to standard manipulation (absent deviant stimulation).

### 2. Average ERPs Reflect Mean Saliency Levels, Irrespective Of Visual Awareness

**Figure 3:**
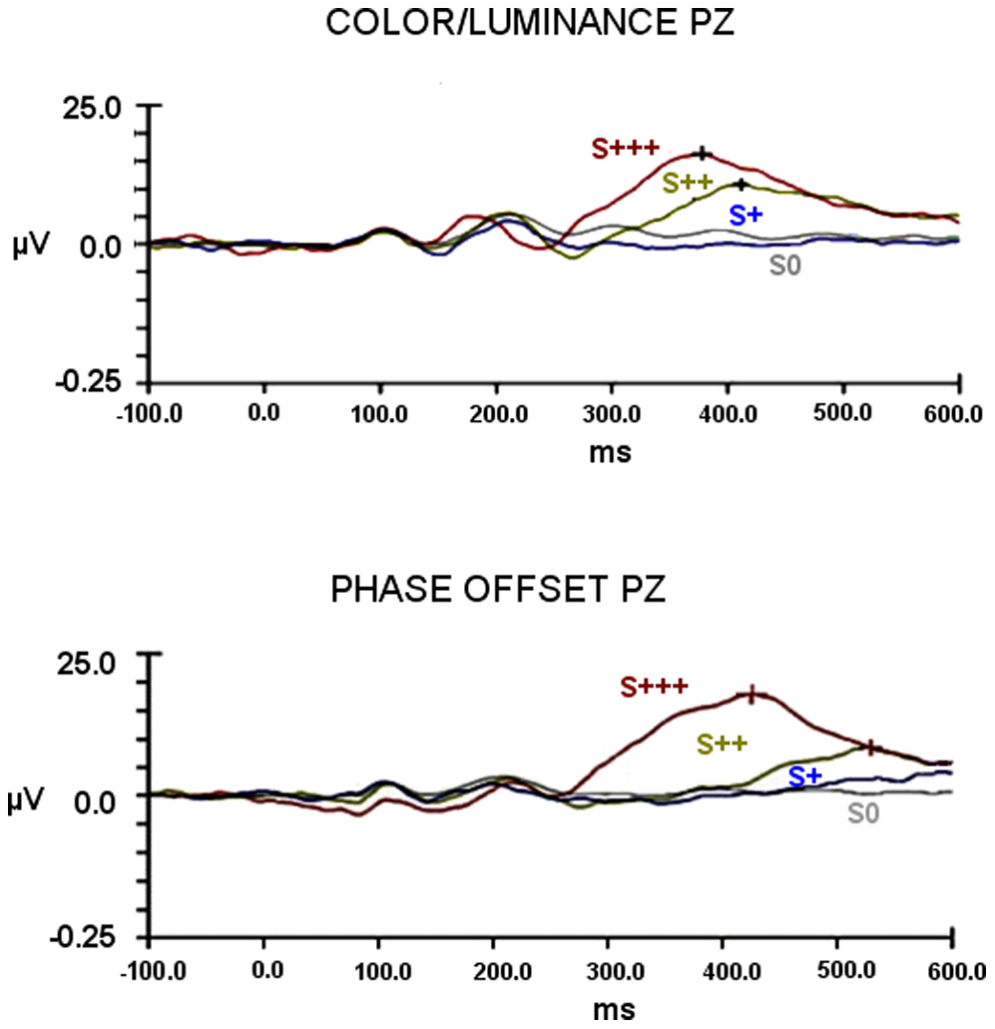
Grand-average ERPs for Experimental Session I (experiments I and II): evidence for saliency related response modulation. Larger and faster responses are evoked by most salient stimuli. Grand-average ERPs', time locked for target category level, are shown for the representative PZ electrode and referenced to earlobes. Amplitude in microvolts (µv) is plotted against time (ms). S0 – ERPs for Standard stimulus; S+ ERPs for minimal saliency manipulation; S++ ERPs for intermediate saliency manipulation; S+++ ERPs for the maximal saliency manipulation. Top: ERP average waveforms for the Color/Luminance experiment. Bottom : ERP average waveforms for the for Phase Offset experiment. Differences between S0 and S+ *average* ERPs were found exclusively for phase offset condition, the only one where stimuli reached conscious access, thereby matching behavioral data (Tables S2 and S7 in File S1; and Fig. 2).

**Figure 4:**
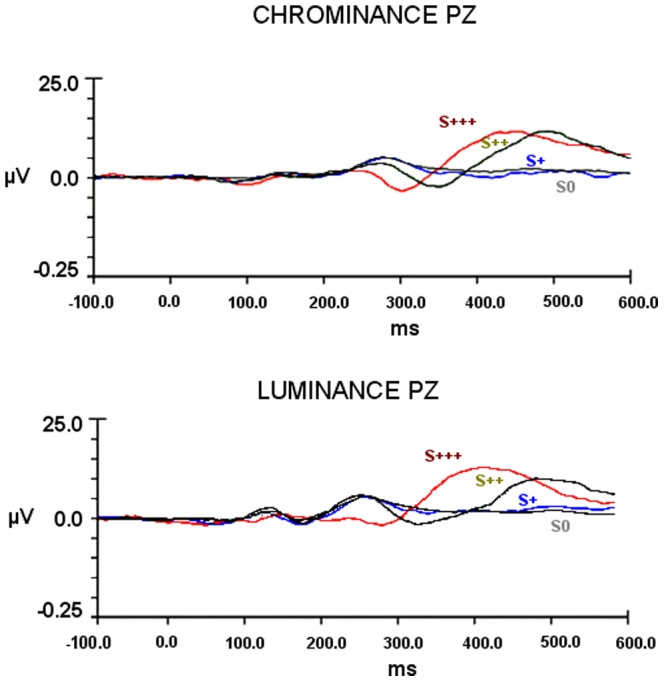
Grand-average ERPs for Experimental Session II (experiments III and IV): Larger and faster responses are confirmed to be present for most salient stimuli. Grand-average ERPs', time locked for target category level are shown for the PZ electrode. Amplitude in microvolts (µv) is plotted against time (ms). S0 – ERPs for Standard stimulus; S+ ERPs for minimal saliency manipulation (none reaching awareness); S++ ERPs for intermediate saliency manipulation; S+++ ERPs for the maximal saliency manipulation. Top - ERP average waveforms for Chrominance experiment. Bottom - ERP average waveforms for Luminance experiment. No differences were found between S0 and S+ evoked *average* ERPs which matches behavioral data (Tables S2 and S7 in File S1; and Fig. 2).

Figures 3 and 4 depict the participants' grand-average ERPs, time-locked for target level at the representative PZ site, for all experiments. We did observe average oddball ERP components to change detection corresponding to P300-like responses. Signal amplitudes where highest for the most salient stimuli and latencies shorter, monotonically changing with the perceptual deviance level from the standard.

#### Experimental Session I

**Figure 5:**
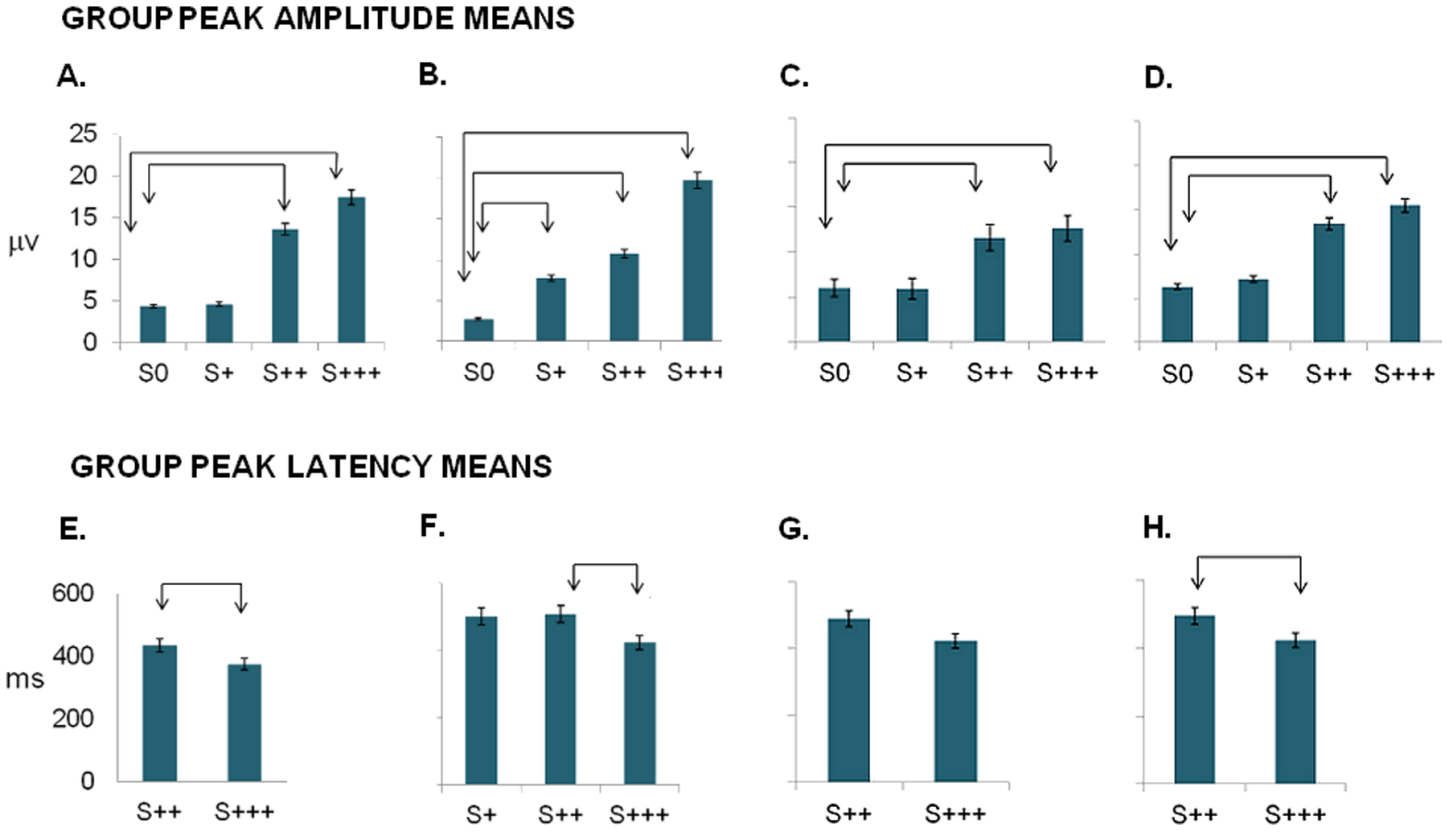
Summary plots of P300 peak amplitude and latency means for averaged ERPs: larger and faster responses for most salient stimuli. Top: A, B, C, E - mean peak amplitudes for A. Color/Luminance (exp I); B. Phase offset (exp II); C. Chrominance (Exp III); D. Luminance (exp IV) evoked average ERPs. Bottom: E, F, G, H - mean peak latencies for E. Color/Luminance (exp I); F Phase Offset (exp II); G. Chrominance (exp III); H. Luminance (exp IV) evoked average ERPs. Amplitudes where highest for the most salient targets, monotonically decreasing along with the physical deviation level (see text for correlation analysis). Significant peak amplitude differences were found between S++ and S+++ saliency levels from Standard S0. No amplitude differences were found between S+ level and Standard S0 *average* ERPs, except for the Phase Offset experiment (matching behavioral data where only in the later condition a behavioral difference was found, e.g. S+ was supraliminal only in this case). Arrow intervals depict significant difference at 0.05. Bars depict confidence intervals for the means at 95%.

We first analyzed responses to low saliency (below threshold in all experiments and near in the Phase Offset experiment) stimuli versus standard. Change detection ERPs for S+ target stimuli were only detectable for phase offset experiment II, which was the only one where conscious reports of target presence were present (see Fig. 3 which shows average waveforms, and also summary peak amplitude and latency measures in Fig. 5, A and B). Accordingly, repeated measures ANOVA showed significant *average* amplitude differences elicited by S+ and S0 within subject responses, only for the Phase Offset experiment [F(_1,9_) = 7.169, *p* = 0.025] (*p*<0.05). Concerning Color/Luminance experiment I, repeated measures ANOVA for Standard S0 and S+ peak *average* amplitudes showed, as expected, no significant effects [F_(1,9)_ = .151, *p* = .706] (*p*<0.05) in line with the absent conscious reports of target presence (Fig. 3 and Fig. 5, A and B).

More stringent within subject analysis effects of overall saliency (sub and supraliminal) levels on signal amplitude relative to Standard, confirmed the above mentioned pattern [Color/Luminance (S0, S+, S++, S+++): F(_3,27_) = 33.1, *p*<0.001; Phase offset(S0, S+, S++, S+++): F(_3,27_) = 29.9, *p*<0.001]. Bonferroni corrected pairwise multiple comparisons confirmed the expected supraliminal S++[(Color/Luminance *p* = 0.003; Phase Offset *p* = 0.003) and S+++(Color/Luminance *p*<0.001; Phase Offset *p*<0.001)] amplitude differences from standard S0. S++ and S+++ amplitudes for phase offset were also significantly different (*p* = 0.009) (Fig. 5, A and B). No differences were seen for subliminal conditions S+ (except the supraliminal phase offset condition).

It is relevant to point out that saliency effects were corroborated by the ranked monotonic effect of physical saliency on response amplitude as shown by the observation of significant Spearman Correlations between saliency levels and neural responses [Color/Luminance *r_s_* = .777(p<0.001); Phase Offset *r_s_* = .775(p<0.001)].

Latencies were systematically shorter for S+++ level than for S++, showing again a saliency effect. Since the ERP component is not detectable on average responses to S+ (subliminal) Color/Luminance condition, latency calculations cannot be obtained for this subliminal saliency level due to the unmeasurable signal (which justifies the bar absence in Figure 5, E, G and H). Repeated measures ANOVA (S++, S+++) analysis of differences in peak latencies was significant for Color/Luminance manipulations [F_(1,9)_ = 15.243, *p* = 0.004 Greenhouse-Geisser corrected]. For Phase Offset conditions, saliency effects on latency were also significant (S+, S++ and S+++ levels) [F_(2,18)_ = 4.136, *p* = 0.033]. Bonferroni corrected pairwise multiple comparisons showed significant differences for S++ and S+++ latencies [*p* = 0.034] (Fig. 5, E and F).

#### Experimental Session II

As with previous experiments I and II (Session I), we confirmed that saliency manipulations modulate neural signal's amplitude and latency, with supraliminal target categories eliciting higher amplitudes at shorter latencies (Fig. 4).

Subliminal S+ target showed no detectable *average* ERP components [Chrominance within subjects' ANOVA S0, S+: F_(1,8)_ = 0.006, *p* = 0.942; Luminance within subjects' ANOVA S0, S+: F_(1,8)_ = .825, *p* = .390) (Fig. 5, C and D).

Analysis of peak amplitude and latencies across all levels did indeed confirm the effects of saliency. Repeated measures ANOVA of within subject amplitude differences was significant for both experiments III and IV [Chrominance F_(3.24)_ = 20.328, *p*<0.001; Luminance F_(3,24)_ = 10.185, *p* = 0.005 Greenhouse-Geisser corrected]. Importantly, Bonferroni corrected pairwise multiple comparisons showed only amplitude differences between supraliminal and standard conditions [Chrominance S++ *p* = 0.016, S+++ *p* = 0.002; Luminance S++*p* = 0.07(marginally sig); S+++ *p* = 0.020] (Fig. 5, C and D). Subliminal stimuli did not elicit significant average responses, as expected.

Spearman correlation analysis confirmed again the observation of a ranked relationship between saliency and signal amplitude: [Chrominance *r_s_* = .772, (p<0.001); Luminance *r_s_* = .769, (p = .002)].

Latencies within experimental session II (experiments III and IV), as explained above for experiments I and II, could not be calculated for subliminal saliency levels, because of the expected failure to detect oddball *average* ERP signals for the S+ subliminal level. Repeated measures ANOVA for S++, S+++ latency differences, were marginally significant for Chrominance [F(1,8) = 5.245, *p* = 0.051 Greenhouse-Geisser corrected] and significant for the Luminance condition [F(1,8) = 6.898, *p* = 0.030 Greenhouse-Geisser corrected] (Fig. 5, G and H).

### 3. Single Trial Classification

Single trial waveform classification uncovered neural events signaling target presence matching conscious reports of perceptual awareness. We could classify target presence both at intraindividual and interindividual levels, and across feature as well as saliency levels, thereby supporting the feasibility of generalization.

Classification performance measures for target detection were obtained through a leave-one-out (LOO) cross validation technique [6], [7] (see Methods). Single Trial classification results are presented in balanced accuracy measures. As explained in the methods section, the balanced accuracy measure is bias free, unlike the “standard accuracy”, which is sensitive to data set imbalances transforming a chance level into a high classification accuracy. This does not happen with the balanced accuracy index, (b_accuracy), that takes into account sensitivity [True Positive_(TP)_/(True Positive+False Negative_(FN)_)] and specificity [True Negative_(TN)_/(True Negative+False Positive_(FP)_)] rates independently of the unbalanced size of the two classes (b_accuracy = (sensitivity+specificity)/2), therefore being a robust and conservative performance index. Nevertheless performance measures of accuracy and precision were also computed (accuracy = (TP+TN/TP+FN+TN+FP); precision = (TP/(TP+FP)). Tables S3, S4, S5 and S6 in File S1, show performance indexes for the two experimental sessions in all computed measures.

#### Experimental Session I

**Figure 6:**
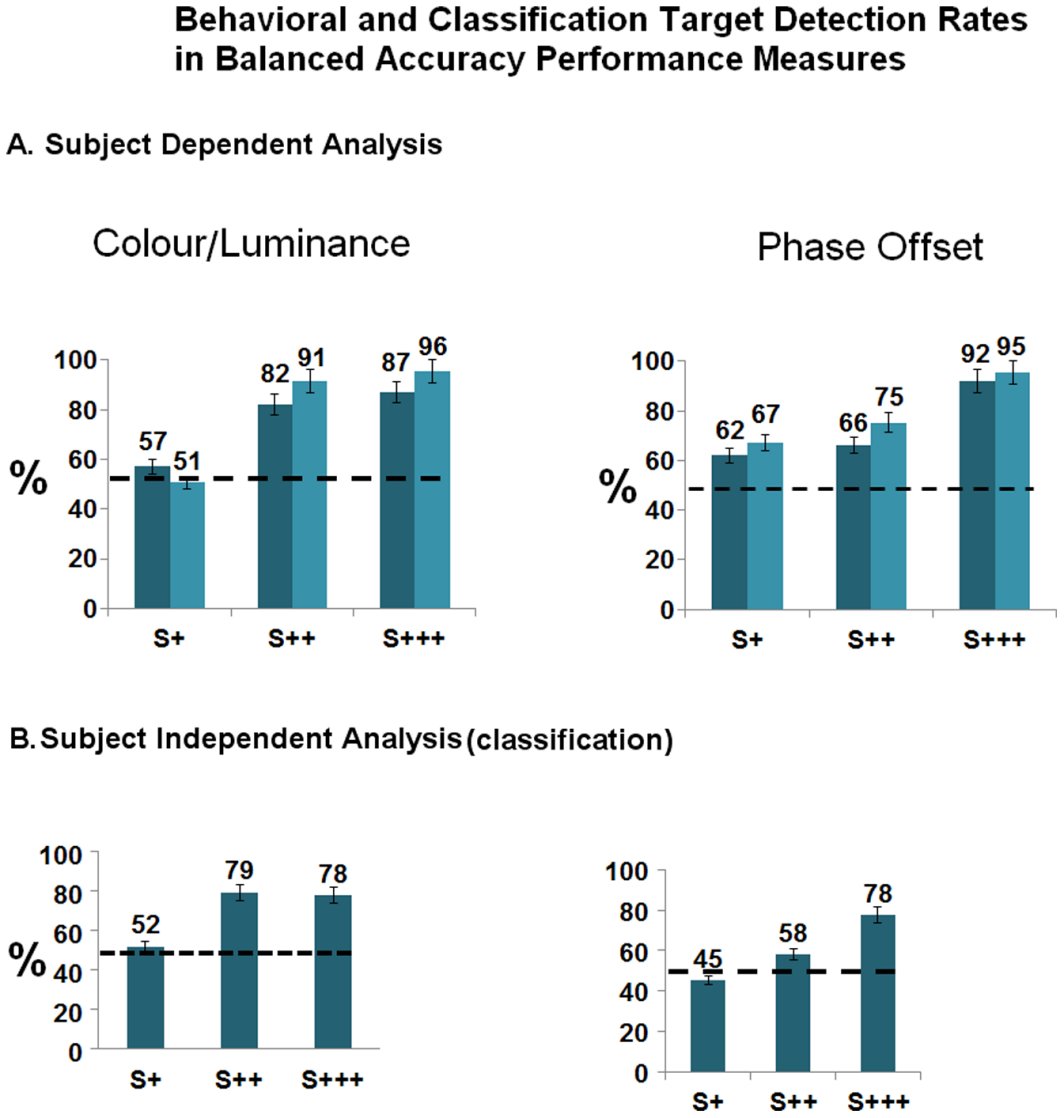
Behavioral and *Single trial* waveform statistical classification performance in terms of (conservative) balanced accuracy – Experiments I & II - single trial statistical classification matches conscious perception and can be generalized across subjects. A. Behavioral and Subject dependent (within subject classification) LOO (leave one out) Classification Balanced Accuracy Rates for Target detection. Right: results for the Color/Luminance experiment. Left: results for the Phase Offset experiment. B. Subject independent (between subject classification) LOO (leave one out) Classification Balanced Accuracy Rates for Target detection. Right: results for the Color/Luminance experiment. Left: results for the Phase Offset experiment. Dashed lines depict chance level at 50%. Bars depict confidence intervals for the means at 95%.

Using the 200–600 ms epoch time-window, balanced accuracy (a very conservative measure, with lower values that simple accuracy, see above and Table S3 in File S1) classification results for waveform target detection amounted to at least 87% to supraliminal targets, 66% for near threshold targets, and 57% for subliminal targets (Table S3 in File S1; and Fig. 6 A).

We then investigated if target detection in a given individual can be used to predict the presence of this response in other individuals. Participants' independent classification models were also used - between subjects' LOO. According to this statistical model, the algorithm for classification is trained using ERP features on data collected from a subset of the most “generalizable” 3 subjects, and tests for target detection in the data of a different subject. It is therefore a simulation of a “universal model” than can be applied to whoever subject's data (model-driven approach). The criterion for generalization was based on classification accuracy metrics and not from the simple perspective of P300 waveform morphology. From the between subjects LOO cross validation, we obtained individual models for each subject, which were tested on all the other subjects. The three models that, on average, performed better on the other subjects, were selected. Then, the final model was learned taking together the respective three datasets. These results suggest that the same neural correlates of target detection can be found not only within but even between subjects (see also additional replication below).

Results for target detection were highest for supraliminal targets (at least 77% balanced accuracy in Phase Offset), followed by near threshold (at least 58%) and near chance level for subliminal stimuli (Table S4 in File S1; and Fig. 6 B. Please note that the apparent below level chance for S+ was a non significant effect that was likely due to random variation).

#### Experimental Session II

**Figure 7:**
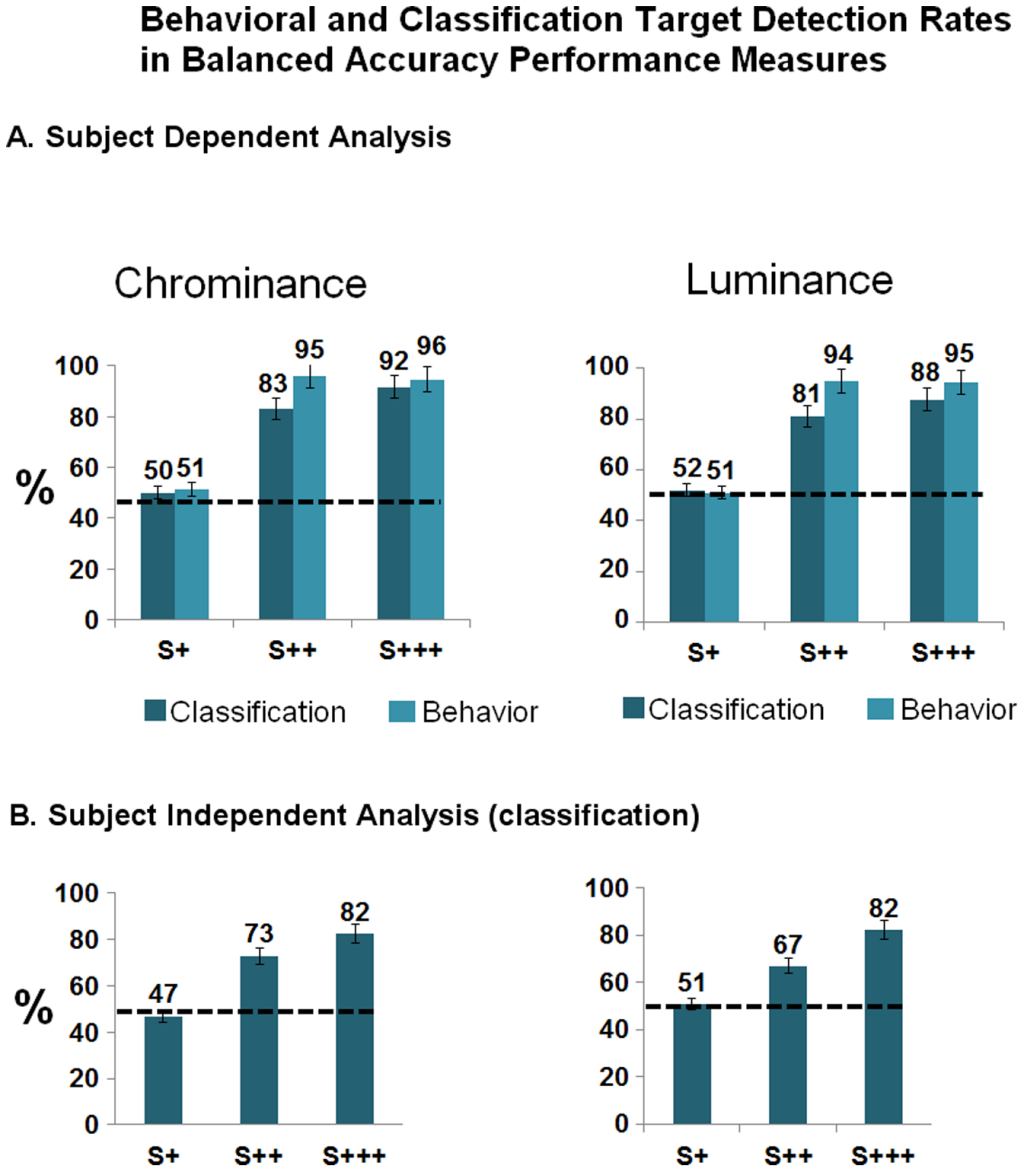
Behavioral and *Single trial* waveform statistical classification performance in terms of (conservative) balanced accuracy – Experiments III & IV – we replicate the finding that single trial statistical classification matches conscious perception and can be generalized across subjects. A. Behavioral and Subject dependent (within subject classification) LOO Classification Accuracy Rates for Target detection. Right: results for the Chrominance experiment. Left: results for the Luminance experiment. B. Subject independent (between subject classification) LOO Classification Accuracy Rates for Target detection. Right: results for Chrominance condition. Left: results the Luminance experiment. Dashed lines depict chance level at 50%. Bars depict confidence intervals for the means at 95%.

Subject dependent (within-individual) classification results for target detection using LOO cross validation model within 200–600 ms epoch window, yielded, in balanced accuracy, target detection rates superior to 87% to supraliminal saliency, to 81% for near-threshold ones and at chance level to subliminally salient stimuli (Table S5 in File S1; and Fig. 7 A). Between subjects cross validation models for classification, yielded waveform target detection rates above 80% for salient supraliminal stimuli, above 67% (at least) for near-threshold ones and at chance levels for subliminal stimuli, (200–600 ms epoch) (Table S6 in File S1; and Fig. 7 B).

**Figure 8:**
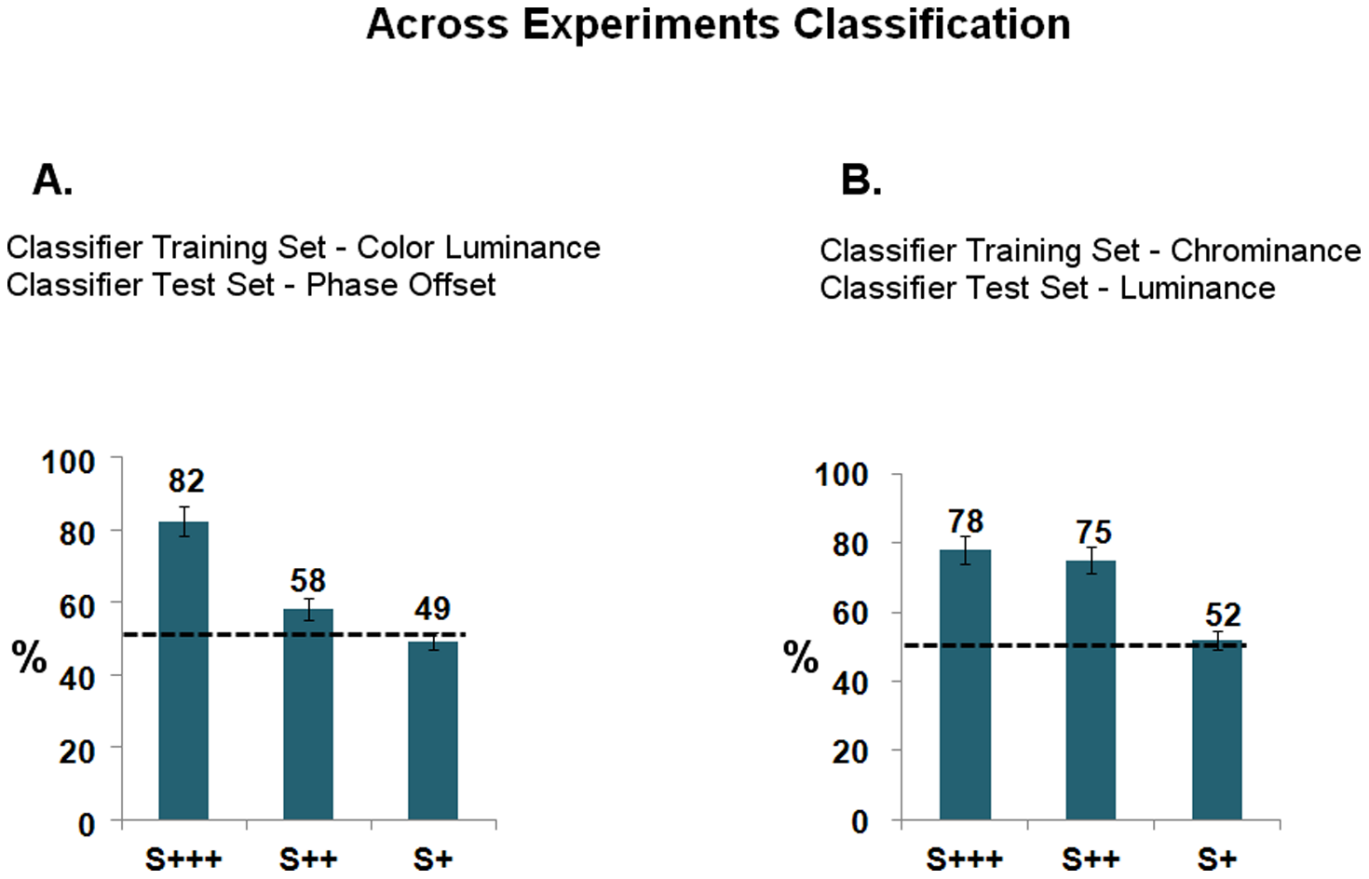
Classification Results Across Experiments, in Balanced Accuracy Performance Measures: classification accuracy is proportional to saliency levels. A. Classification model fot target detection built from the Color Luminance experiment and used to classify Phase Offset experiment events'. B. Classification modelfor target detection built from the Chrominance experiment and used to classify Luminance experiment events'. Dashed lines depict chance level at 50%. Bars depict confidence intervals for the means at 95%.

Two additional analyses were then made to test this generalization across conditions (Fig. 8). In the first one, the classification model was built from the Color/Luminance experiment, and then the model was used to classify Phase Offset events. In the second one, the classification model was built from the Luminance experiment, and then the model was used to classify Chrominance events.

**Figure 9:**
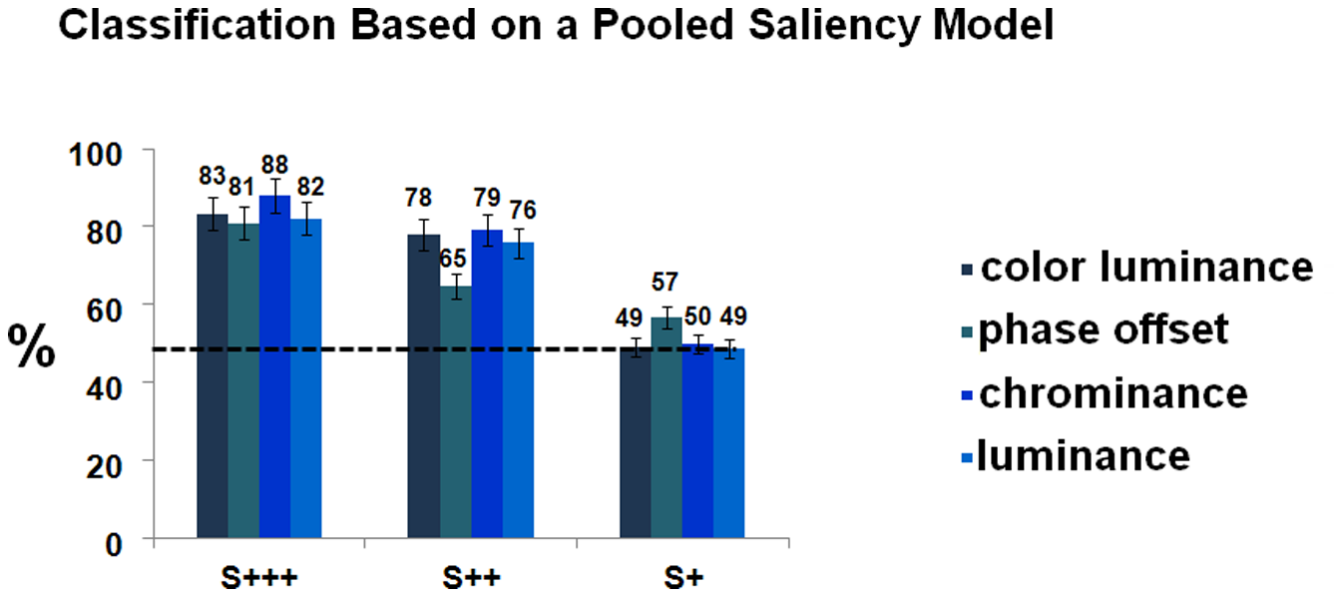
Classification Results Based on a Pooled Saliency Model, in Balanced Accuracy Performance Measures: classification accuracy is proportional to saliency levels. A classification model was built from a pool of all S+/S++/S+++ target responses, and used to classify the events of each experiment. Dashed lines depict chance level at 50%. Bars depict confidence intervals for the means at 95%.

Figure 9 shows results from a model that was learned taking the pool of S+++/S++/S+ target responses, and then used for classification. Classification even improves using this more general approach. We also looked for a specific decoding performance rate by saliency level. A model was designed training for each particular saliency level and testing over each of the others. If a general marker of conscious perception is the most likely hypothesis at stake, the prediction would be the classifier to fail target detection for the S+ (feature extraction) based model, being the learning driven by the supraliminal evoked related activity (S++ and S+++ based models). The results matched the prediction, the classifier being able to detect the target presence significantly and well above chance only for the graded supraliminal saliency levels models (with performance rates up to ∼90%).

## Discussion

It this study we found evidence for a general conscious perception signal which can be robustly “read out” across experiments and even subjects. These results have implications both concerning the potential use in brain computer interfaces and the cognitive neuroscience of sensory awareness. Concerning the former, the observed across-individual generalization is promising for use in BCI applications because training of the classifier becomes much simplified and accelerated due to the between-subjects common neurophysiological features. Our findings suggest that variability of individual brain signatures of each BCI user may actually be overcome in such a way that it relieves from the need of extensive user training [28]. This suggests that it is possible to perform BCI experiments under our conditions with significant reduction of calibration measurements and without loss of classification performance. This offers the potential to surpass methods taking advantage of knowledge collected in previous sessions. Generalization to independent runs more safely ensures independence of the test set, and overcomes a much more challenging goal than generalization to novel stimuli within the same or similar categories.

The neuroscientific implications of our findings are also interesting because they suggest that a common neural signature of visual awareness is present across individuals. This finding suggests that its cortical generators are quite consistent across subjects even upon detection of oddball visual targets of variable saliency. This is in line on the tenet of Linden 2005, on the consistency of the multiple generators of P300 signals [29].

The actual physical saliency of the stimulus is closely related to the probability of conscious perception and its detection at the single trial level. Neither detection nor perception modulation was present for minimal deviance (subliminal) targets. The question remains whether one can establish a relation between all or none conscious perception and graded perceptual saliency given that the P300 is closely related to visual awareness at the single trial level. “All or none” processes at the single cell level are well known to take the form of a continuous process at the population level. In the case of our experiment, two alternative possibilities can be considered: 1. the “single trial P300” has fixed amplitude across single detection trials and is null in non detection trials (and would therefore be an “all or none” in the same sense that the action potential is classified as “all or none” in spite of being also a continuous process; 2. the P300 has variable amplitude across single trials. Our data are more in favor of the first possibility given the tight link identified between behavioral detection and P300 detection, and virtually “null” average P300 for subliminal S+ stimuli (and maximal for S+++). We would like to emphasize that this link is indirect and speculative. Although it is clear that the P300 is virtually null together with absent detection for S+ (the “none” aspect of the response), it is still debatable if the maximal response is fixed (although this is likely given that it increases proportionally with detection rates to a maximum at S+++). In sum, our results are consistent with the notion that saliency levels modify the probability of all or none conscious perception events and neural responses, in line with other accounts [30]–[32].

We could accurately classify well above chance single perceptual events with across subject, across experiment, and across saliency generalization. The ultimate approach assumed that conscious perception can be decoded as such independently of stimulus salience, provided it is supraliminar. We performed the decoding procedure on all the S+/S++/S+++ data pooled together and found a significant global decoding performance rate. We also found that the specific decoding performance rate confirms the expectation that the decoder fails to extract information from subliminal stimuli (S+), the learning being largely driven by the neurophysiological signatures of supraliminar stimuli S++ and S+++.

These findings, as stated above, pave the way for novel brain computer interfaces that are able to track conscious perception and train cognitive domains such as attention. These achievements were possible through the identification of neurophysiological signatures of conscious perception at the level of single sensory events, attainable because our statistical tools were powerful enough to be used at the single trial level. Single trial based statistics enabled a direct access to visual awareness that cannot be achieved by simply measuring average responses. It is important in this respect to discuss what could be the origin of the achieved classification performance, in particular in which concerns the cortical origin of the P300-like signals, and the possible influence of spatial and feature attention on variability of “cortical” signals. Other issues concern the possible influence of eye movements, that are unlikely given the correction procedures adopted (see Methods). We recently addressed the issue of gaze independence and covert attention by developing a novel P300-based gaze independent paradigm and proving that subjects can effectively control the BCI interface without moving the eyes (using covert attention). ([33], see also [34]). It is also worth pointing out that feature-selective modulation of P300 amplitudes tends to be limited to the attended location [35]. In general, the pattern of modulation may be quite complex due the known pattern of generators concerning target-related responses including the parietal and the cingulate cortices. Novelty-related activations occur mainly in the inferior parietal and prefrontal regions (target frequency modulating their amplitude) and visual modality-specific contributions come from the inferior temporal and superior parietal cortex (for a review see [29], [36]). Importantly, both higher visual and supramodal association areas contribute to the visual P3b pointing to the involvement of distinct attentional subsystems in the detection of rare events [37]. Our finding that conscious perception is associated with modulation of the P3 component (mostly P3b) is in line with the outcomes of other previous studies [9], [14], [30], [31], [38]–[44]. In sum, *global decoding performance reflects conscious perception events since at all saliency levels it matches conscious reports.*

P300, attention and visual awareness are unequivocally related concepts, and this relation has been extensively reviewed [1], [45]–[48]. In any case, seen and unseen stimuli all had the same attentional (spatial and non-spatial) and task demands.

The idea of this study was not to develop a new P300 paradigm but rather to interrogate it at as tool to explore perceptual saliency and conscious perception. In fact it has been controversial whether the P300 solely reflects conscious processing because most approaches were model driven (see introduction) and our data driven approach now settles this question, by showing a close match between conscious detection and P300 detection at the single trial level. The observed reliable detection was rendered possible by using our previously validated single trial classification strategies that were successfully applied in BCI applications [6], [7]. Using such techniques, we were able to provide a direct link between psychophysics and neurophysiology, and to find evidence for visual awareness, even when using conservative balanced accuracy measures.

The power of pattern-classification methods has been shown in the domain of fMRI, and in the vision domain it has been shown that conscious perception of particular contexts can be detected in brain representations of nonstimulated regions [49]. Moreover, brain reading of fMRI signals has also been used to identify activation patterns that encode the perceptual interpretation of ambiguous stimuli irrespective of their physical characteristics [50]. However temporal resolution of such pattern recognition techniques is rather low and requires the integration of many perceptual moments. This study is novel because it investigates online visual awareness and conscious perception at a temporal resolution much higher the one available using fMRI, allowing for parsing single sensory events at different saliency levels.

In the domain of EEG to our knowledge, there are no available studies of single trial EEG brain reading of percepts of variable saliency and the neural correlates of visual awareness. A previous study [51] addressed the prediction of *erroneous* single trial *conscious* percepts. A former study on brain activity based image classification from rapid serial visual presentation, proposed the use of this concept for BCI applications [52]. Other studies have attempted the prediction of EEG single trial responses from other signals such as concomitant fMRI [53] but not predicting correct perception from such responses. Although a single-trial analytic framework for EEG analysis has been proposed for target detection and classification [54], and application in BCI interfaces [6], [7] no study before searched for direct event related correlates of conscious perception at different saliency levels.

Our findings do therefore suggest a novel approach for direct neurophysiological detection of single sensory events irrespective of their perceptual conspicuity. In other words, regardless of whether a object pops out effortlessly or not in our visual experience, it can be identified at the neural level regardless of whether it differs markedly from the surround or not. To our knowledge such a correlate of visual awareness irrespective of saliency at the EEG single trial level was not reported before in the field of conscious perception.

It is worth noting that a few previous studies using oddball paradigms also using threshold/subtreshold stimulation [15]–[17] suggested that subthreshold stimulation enhanced P300 suprathreshold oddball responses. However, other studies suggest that this average measure is strictly associated with conscious, explicit perception [8], [9], [11], [13], [14], [30], [31], [38]–[43]. Our study provides clear evidence favoring the latter view because it provides direct evidence that single trial waveforms that overlap with the timing of P300 components are directly related to conscious detection at the single trial level.

In sum, we identified neurophysiological evidence for single trial sensory representations and visual awareness at different saliency levels. This neural correlate of conscious perception is robustly detected for these unique sensory events, both within and between subjects, using brain reading techniques. We conclude that neural signatures of *single trial* perceptual conscious perception can be found irrespective of their perceptual strength. Indeed, conscious perception can be decoded as such independently of stimulus salience. This study therefore provides a new framework to study online visual awareness and conscious perception at high temporal resolution.

The possibility to directly undercover stimulus event's related conscious awareness showed surprising fidelity even when using conservative balanced accuracy measures. The use of single trial statistical classification showed that one can detect single fleeting events reaching visual awareness at the intraindividual as well as at the interindividual level, paving the way for studies of conscious perception that are truly based on single events.

## Supporting Information

File S1 **Table S1, Guessing rate estimation for each experiment. Table S2, Group statistics of Target detection proportion against guessing rate. Table S3, Single Trial Classification Performance Measures – Experimental Session I. Table S4, Single Trial Classification Performance Measures – Experimental Session I. Table S5, Single Trial Classification Performance Measures – Experimental Session II. Table S6, Single Trial Classification Performance Measures – Experimental Session II. Table S7, Individual guessing rates and one sample binomial tests to S+ binary detection proportion.**
(DOC)
