# Supplementary material for: Robust Single Trial Identification of Conscious Percepts Triggered by Sensory Events of Variable Saliency
Source: PLoS One. 2014 Jan 23;9(1):e86201. doi: 10.1371/journal.pone.0086201 (PMC3900484; doi:10.1371/journal.pone.0086201)
Supplement: File S1 — Table S1, Guessing rate estimation for each experiment. Table S2, Group statistics of Target detection proportion against guessing rate. Table S3, Single Trial Classification Performance Measures – Experimental Session I. Table S4, Single Trial Classification Performance Measures – Experimental Session I. Table S5, Single Trial Classification Performance Measures – Experimental Session II. Table S6, Single Trial Classification Performance Measures – Experimental Session II. Table S7, Individual guessing rates and one sample binomial tests to S+ binary detection proportion. (DOC) [file pone.0086201.s001.doc]

**SUPLEMENTAL DATA-1**

**Table S1. Guessing rate estimation for each experiment.**

Standard stimuli N for experimental session I was 8930. Standard stimuli N for experimental session II was 8460. Guessing rates proved to be similar to probability of target occurrence.

| Experiments | False Positive (FP) responses to Standard | True Negative  (TN) responses to Standard | Total  (Standard N) | Guessing rate (FP/(FP+TN)) |
| --- | --- | --- | --- | --- |
| Color/Luminance | 416 (5%) | 8514 (95%) | 8930 | 0.05 (5%) |
| Phase Offset | 232 (3%) | 8698 (97%) | 8930 | 0.03 (3%) |
| Chrominance | 432 (5%) | 8028 (95%) | 8460 | 0.05 (5%) |
| Luminance | 456 (5%) | 8004 (95%) | 8460 | 0.05 (5%) |

**Table S2. Group statistics of Target detection proportion against guessing rate.**

True positive target detection (S+/S++/S+++) results for each experiment were tested against guessing rate. No differences were found between S+ and salience S0 standard detection proportions, except for the phase offset experiment II. Levels S++ and S+++ were significantly different from S0 for all experiments.

| Experimental Saliency levels | True Positive | False Negative | Guessing Rate  (Test proportion for binomial non-parametric test) | Statistical significance (p value) |
| --- | --- | --- | --- | --- |
| Color/Luminance **S+** | 11 (**6%;**n=190) | 179 (94%) | 0.05 (5%) | **ns** |
| Phase offset **S+** | 70 (**37%;**n=190) | 120 (63%) | 0.03 (3%) | **0.00** |
| Chrominance **S+** | 14 (**8%;** n=180 | 166 (92%) | 0.05 (5%) | **ns** |
| Luminance **S+** | 13 (**7%;** n=180 | 167 (93%) | 0.05 (5%) | **ns** |
| Color/Luminance **S++** | 166 (**87%;**n=190**)** | 24 (13%) | 0.05 (5%) | **0.00** |
| Phase offset **S++** | 105 (**55%**n=190) | 85 (45%) | 0.03 (3%) | **0.00** |
| Chrominance **S++** | 164 (**97%;**n=180) | 6 (3%) | 0.05 (5%) | **0.00** |
| Luminance **S++** | 171(**95%;**n=180) | 9 (5%) | 0.05 (5%) | **0.00** |
| Color/Luminance **S+++** | 182 (**96%;** n=190) | 8 (4%) | 0.05 (5%) | **0.00** |
| Phase offset **S+++** | 181 (**95%;**n=190) | 9 (5%) | 0.03 (3%) | **0.00** |
| Chrominance **S +++** | 170 (**94%;**n=180) | 10 (6% | 0.05 (5%) | **0.00** |
| Luminance **S+++** | 169 (**94%;**n=180 | 11 (6% | 0.05 ( 5%) | **0.00** |

**Tables S3 and S4. Single Trial Classification Performance Measures – Experimental Session I.**

Session I subject dependent – S53 (within subject), and independent – S4 (between subject) classifier performance measures, within the 200-600 ms time window epochs, through LOO cross validation technique. Note the high single trial performance, even for corrected measures such as balanced accuracy, and conservative approaches such as user independent (between subject) classification.

S3.

| Subject Dependent Single Trial Classification Measures Epoch Time-window: 200-600 ms | | | | | | |
| --- | --- | --- | --- | --- | --- | --- |
| Color/Luminance | S levels | **accuracy** | **b_accuracy** | **sensitivity** | **specificity** | **precision** |
|  | S+++ | 0.9353 | 0.8688 | 0.7994 | 0.9382 | 0.2378 |
|  | S++ | 0.9155 | 0.8166 | 0.7133 | 0.9198 | 0.1953 |
|  | S+ | 0.8486 | 0.5706 | 0.2806 | 0.8606 | 0.0422 |
| Phase Offset | S+++ | 0.9523 | 0.9180 | 0.8822 | 0.9538 | 0.3161 |
|  | S++ | 0.8719 | 0.6581 | 0.4350 | 0.8812 | 0.0775 |
|  | S+ | 0.8432 | 0.6236 | 0.3944 | 0.8528 | 0.0582 |

S4.

| Subject Independent Single Trial Classification Measures Epoch Time-window: 200-600 ms | | | | | | |
| --- | --- | --- | --- | --- | --- | --- |
| Color/Luminance | S levels | accuracy | b_accuracy | sensitivity | specificity | precision |
|  | S+++ | 0.862 | 0.781 | 0.695 | 0.649 | 0.104 |
|  | S++ | 0.832 | 0.794 | 0.644 | 0.836 | 0.080 |
|  | S+ | 0.626 | 0.519 | 0.409 | 0.630 | 0.023 |
| Phase Offset | S+++ | 0.898 | 0.776 | 0.649 | 0.903 | 0.129 |
|  | S++ | 0.794 | 0.582 | 0.362 | 0.803 | 0.036 |
|  | S+ | 0.649 | 0.453 | 0.249 | 0.657 | 0.016 |

**Tables S5 and S6. Single Trial Classification Performance Measures – Experimental Session II.**

Session II subject dependent – S5 (within subject), and independent – S6 (between subject) classifier performance results, within the 200-600 ms time window epochs, through LOO cross validation technique. We replicate the high performance of single trial classifiers.

S5.

| Subject Dependent  Epoch Time-window: 200-600 ms | | | | | | |
| --- | --- | --- | --- | --- | --- | --- |
| Chrominance | S levels | **accuracy** | **b_accuracy** | **sensitivity** | **specificity** | **precision** |
|  | S+++ | 0.9525 | 0.9170 | 0.8800 | 0.9540 | 0.3245 |
|  | S++ | 0.9094 | 0.8314 | 0.7500 | 0.9129 | 0.1662 |
|  | S+ | 0.8190 | 0.4991 | 0.1650 | 0.8331 | 0.0213 |
| Luminance | S+++ | 0.9402 | 0.8765 | 0.8100 | 0.9430 | 0.2723 |
|  | S++ | 0.9258 | 0.8104 | 0.6900 | 0.9309 | 0.1952 |
|  | S+ | 0.8416 | 0.5203 | 0.1850 | 0.8555 | 0.0284 |

S6.

| Subject Independent Single Trial Classification Measures Epoch Time-window: 200-600 ms | | | | | | |
| --- | --- | --- | --- | --- | --- | --- |
| Chrominance | S levels | **accuracy** | **b_accuracy** | **sensitivity** | **specificity** | **precision** |
|  | S+++ | 0.813 | 0.824 | 0.836 | 0.812 | 0.098 |
|  | S++ | 0.692 | 0.728 | 0.764 | 0.691 | 0.052 |
|  | S+ | 0.759 | 0.468 | 0.164 | 0.772 | 0.015 |
| Luminance | S+++ | 0.816 | 0.822 | 0.829 | 0.816 | 0.093 |
|  | S++ | 0.780 | 0.671 | 0.557 | 0.785 | 0.054 |
|  | S+ | 0.782 | 0.508 | 0.221 | 0.794 | 0.022 |

**Note to tables S3/S4/S5/ S6:** Given the imbalance between the two classes, the precision measure (TP/(TP+FP) is uninformative. A simple example renders clear why this is the case: each target saliency has a probability of 20/940 (20 targets / 940 standards). If 10% of the targets are detected as standards and if 10% of the standards are detected as targets, there will be 18 TP, 2 FN, 846 TN and 94 FP. This would result on 90% balanced accuracy, 90% standard accuracy, 90% sensitivity, 90% specificity, and only 0.16 precision (18/(18+94)). Balanced accuracy is the appropriate measure to assess the classification performance, which corresponds to the arithmetic mean of sensitivity and specificity. The performance measures of standard accuracy and precision presented in Tables S3/S4/S5/S6/ are only provided as a complement.

**Table S7. Individual guessing rates and one sample binomial tests to S+ binary detection proportion.**

Participant individual guessing rates were calculated and used as test value for one sample binomial tests against S+ target detection proportion. Group results are summarized per experiment. Except for phase offset , no differences were found between S+ and Standard S0 individual detection proportions, with most participant results retaining the null hypothesis that hit (true positives) and miss (false negatives) categories occurred within the estimated probability

| **Experimental Session I - Color/Luminance** | | | | |
| --- | --- | --- | --- | --- |
| Subjects | False Positives’  frequency  to Standard | False Positive rate  to Standard | S+ detection frequency | One Sample  Binomial Test  (ALPHA=0.05)  SUCCESS CATEGORICAL=1  H0=success proportion occurs within the hypothesized probability  H1= success proportion differs from the hypothesized probability |
| S1 | 2(0.2%;n=940) | 0.002 | 0 | .961(TESTVALUE=0.002) |
| S2 | 1(0.1%; n=846) | 0.001 | 0 | .982(TESTVALUE=0.001) |
| S3 | 0 (n=846) | 0 | 0 | --- |
| S4 | 1(0.1%;n=940) | 0.001 | 0 | .980(TESTVALUE=0.001) |
| S5 | 0(n=846) | 0 | 0 | --- |
| S6 | 168(2%;n=940) | 0.2 | 5(3%) | .370(TESTVALUE=0.2) |
| S7 | 1(0.1%;n=846) | 0.001 | 0 | .982 (TESTVALUE=0.001) |
| S8 | 0(n=940) | 0 | 0 | --- |
| S9 | 241(3%;n=940) | 0.3 | 6(3%) | .541(TESTVALUE=0.3) |
| S10 | 2(0.2%; n=846) | 0.002 | 0 | .965 (TESTVALUE=0.002) |
| ***TOTAL*** | ***416  (5%;n=8930)*** | ***0.05*** | ***11 (6%;n=190; FN=179;94%)*** | ***0.370 (TESTVALUE=0.05)*** |
| **Experimental Session I Phase Offset** | | | | |
| S1 | 10(1%; n=940) | 0.01 | 14(70%; n=20) | *0.00 (TESTVALUE=0.01)* |
| S2 | 9(1%; n=846) | 0.01 | 15(83%; n=18) | *0.00(TESTVALUE=0.01)* |
| S3 | 8(0.9%;n=846) | 0.009 | 0 (n=18) | 0.850(TESTVALUE=0.009) |
| S4 | 3(0.3%;n=940) | 0.003 | 0(n=20) | 0.942(TESTVALUE=0.003) |
| S5 | 8(0.9%; n=846) | 0.009 | 1(6%; n=18) | 0.150(TESTVALUE=0.009) |
| S6 | 28(3%; n=940) | 0.03 | 16 (80%;n=20) | *0.00(TESTVALUE=0.02)* |
| S7 | 1(0.1%;n=846) | 0.001 | 0 (n=18) | 0.982 (TESTVALUE=0.001) |
| S8 | 1(0.1%; n=940) | 0.001 | 6 (30%;n=20) | *0.00 (TESTVALUE=0.001)* |
| S9 | 163(20%;n=940) | 0.2 | 18 Total (90%;n=20) | *0.00 (TESTVALUE=0.2)* |
| S10 | 1(0.1%; n=846) | 0.001 | 0 (n=18) | 0.982 (TESTVALUE=0.001) |
| ***TOTAL*** | ***213 (3%; n=8930)*** | ***0.03 (n=8930)*** | ***70***  ***(37%;n=190; FN=120;63%)*** | ***0.00 (TESTVALUE=0.03)*** |

| **Experimental Session II - Chrominance** | | | | |
| --- | --- | --- | --- | --- |
| Subjects | False Positives´  Frequency to Standard | False Positive rate  to Standard | S+ detection frequency | One Sample  Binomial Test  (ALPHA=0.05)  SUCCESS CATEGORICAL=1  H0=success proportion occurs within the hypothesized probability  H1= success proportion differs from the hypothesized probability |
| S1 | 25(3%;n=940) | 0.03 | 1 (5%;n=20) | 0.456(TESTVALUE=0.03) |
| S2 | 21(2%;n=940 | 0.02 | 0 | 0.668(TESTVALUE=0.02) |
| S3 | 19 (2%;n=940) | 0.02 | 1 (5%;n=20) | 0.332(TESTVALUE=0.02) |
| S4 | 29(3%;n=940) | 0.03 | 0 | 0.544(TESTVALUE=0.03) |
| S5 | 19(2%;n=940) | 0.02 | 1(5%,n=20) | 0.332(TESTVALUE=0.02) |
| S6 | 29(3%;n=940) | 0.03 | 0 | 0.544(TESTVALUE=0.03) |
| S7 | 41(4%;n=940 | 0.04 | 0 | 0.442(TESTVALUE=0.04) |
| S8 | 19(2%;n=940) | 0.02 | 0 | 0.668(TESTVALUE=0.02) |
| S9 | 230(20%;n=940) | 0.2 | 11(60%;n=20) | *0.00(TESTVALUE=0.2)* |
| ***TOTAL*** | ***432(5%;n=8460)*** | ***0.05*** | ***14(8%;n=180;FN=166;92%)*** | ***0.336(TESTVALUE=0.05)*** |
| **Experimental session II - Luminance** | | | | |
| S1 | 27(3%;n=940) | 0.03 | 0 | 0.544(TESTVALUE=0.03) |
| S2 | 27(3%;n=940) | 0.03 | 0 | 0.544(TESTVALUE=0.03) |
| S3 | 21(2%%;n=940) | 0.02 | 0 | 0.668(TESTVALUE=0.02) |
| S4 | 31(3%;n=940) | 0.03 | 0 | 0.544(TESTVALUE=0.03) |
| S5 | 21(2%%;n=940) | 0.02 | 0 | 0.338(TESTVALUE=0.02) |
| S6 | 31(3%;n=940) | 0.03 | 4(20%;n=20) | *0.03(TESTVALUE=0.03)* |
| S7 | 43(5%;n=940) | 0.05 | 0 | 0.358(TESTVALUE=0.05) |
| S8 | 21(2%,n=940) | 0.02 | 0 | 0.668(TESTVALUE=0.02) |
| S9 | 234(20%;n=940) | 0.2 | 9(45%;n=20) | *0.01(TESTVALUE=0.2)* |
| ***TOTAL*** | ***456(5%;n=8460)*** | ***0.05*** | ***13(7%;n=180; FN=167;93%)*** | ***0.116(TESTVALUE=0.05)*** |
